# Contact characteristics and factors associated with the degree of urgency among older people in emergency primary health care: a cross-sectional study

**DOI:** 10.1186/s12913-020-05219-0

**Published:** 2020-04-22

**Authors:** Lisa Marie Haraldseide, Linn Solveig Sortland, Steinar Hunskaar, Tone Morken

**Affiliations:** 1 National Centre for Emergency Primary Health Care, NORCE Norwegian Research Centre, Kalfarveien 31, NO-5018 Bergen, Norway; 2 Department of Global Public Health and Primary Care, University of Bergen, Bergen, Norway

**Keywords:** Aged, Emergency medical service (EMS), International classification of primary care (ICPC), Norway, Older people, Out-of-hours, Prehospital, Primary health care, Reason for encounter, Triage

## Abstract

**Background:**

As the proportion of older people increases, so will the consumption of health services. The aim of this study was to describe the contact characteristics among older people and to identify factors associated with the degree of urgency at the Norwegian out-of-hours (OOH) emergency primary health care services.

**Methods:**

Inhabitants aged ≥70 years who contacted the OOH service during 2014–2017 in seven OOH districts in Norway were included. We investigated the variables sex, age, time of contact, mode of contact, ICPC-2 based reason for encounter (RFE), priority degree and initial response. We also performed frequency analyses, rate calculations and a log-binomial regression.

**Results:**

A total of 38,293 contacts were registered. The contact rate/1000 inhabitants/year was three times higher in the oldest age group (≥90 years) compared to the youngest age group (70–74 years). Direct attendance accounted for 8.4% of the contacts and 32.8% were telephone contacts from health professionals. The most frequent RFE chapter used was “A General and unspecified” (21.0%) which also showed an increasing rate with higher age. 6.0% of the contacts resulted in a home visit from a doctor. Variables significantly associated with urgent priority degree were RFEs regarding cardiovascular (Relative risk (RR) 1.85; CI 1.74–1.96), neurological (RR 1.55; CI 1.36–1.77), respiratory (RR 1.40; CI 1.30–1.51) and digestive (RR 1.22; CI 1.10–1.34) issues. In addition, telephone calls from health professionals (RR 1.21; CI 1.12–1.31), direct attendance (RR 1.13; CI 1.04–1.22), contacts on weekdays (RR 1.13; CI 1.06–1.20) and contacts from men (RR 1.13; CI 1.09–1.17) were significantly associated with urgent priority degree.

**Conclusions:**

This study provides important information about the Norwegian older inhabitants’ contact with the OOH emergency primary health care services. There are a wide variety of RFEs, and the contact rate is high and increases with higher age. Telephone contact is most common. The OOH staff frequently identify older people as having “general and unspecified” reasons for encounters. OOH nursing staff would benefit from having screening tools and enhanced geriatric training to best support this vulnerable group when these individuals call the OOH service.

## Background

According to reports from the World Health Organization, the proportion of older people is increasing worldwide [1]. In 2018, 12% of the Norwegian population were 70 years or older, with an expected increase to 18% in 2040 [2]. Apart from young children, older people are among the most frequent users of emergency primary health care services [3–8].

Older patients often have multiple health problems, generally entailing polypharmacy. They are also more likely to have cognitive and functional impairments. Frailty is a common geriatric syndrome among older persons and increases in prevalence with higher age. It is related to physiological changes and a reduced reserve capacity, making older frail people vulnerable to stressors like infections, acute illness or injuries [9]. Altered vital signs represent challenges in clinical assessment among these patients [10]. Furthermore, older patients often present with diffuse symptoms or an atypical presentation of acute illness or trauma, which makes them vulnerable for possible undertriage, delayed evaluation and worsened outcome [11–19]. Another characteristic of older patients is the reluctance to seek help when needed [20]. Worries about their perception of the degree of urgency being appropriate, and about travelling at night are frequently barriers that make older people hesitant about using out-of-hours (OOH) services, even when such use is necessary [21].

**Fig. 1:**
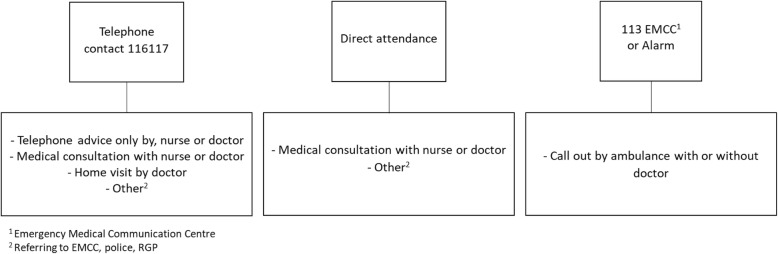
Flow chart describing the potential alternatives to contact the OOH emergency primary health care centre and all possible responses the OOH centre can initiate

In 2019, a national quality reform for an age-friendly Norway was implemented. The goal of this reform is to contribute to better health, quality and mastery of life along with improved years of life. The reform also emphasizes that the older people should receive the health care they need [22], including home care and nursing home services, rehabilitation, regular general practitioner (RGP) and OOH emergency primary health care services [23]. The OOH services are organized differently between countries. Some countries allow direct access to hospital-based care for emergencies, while other countries have a more restricted access [24]. In Norway, the primary health care service has a gatekeeper-function and patients may not go directly to hospital without a medical referral. The municipalities are responsible for the organization of the emergency primary health care 24 h a day. During daytime, patients in need of medical assistance are expected to consult RGPs. Outside opening hours, RGPs take turns working at the local OOH centre, which in some cases is organized within the individual municipality or in joint cooperation among multiple municipalities [25, 26]. The OOH services in Norway have a national telephone number (116117), enabling one to call and talk to an OOH nurse. In some places, one can go directly to the OOH centre without giving advance notice. Based on the reason for encounter (RFE) and degree of severity, the OOH nurse decides which response to initiate (Fig. 1).

Previous studies have reported that the most frequent RFEs resulting in telephone advice among older people are “concerns about/fear of medical treatment”, “general symptoms” and “shortness of breath/dyspnoea” [27]. Furthermore, a European study reported that the most frequent chapters of ICD-10 diagnosis concerning people 65 years or older in Norway were chapter “L Musculoskeletal”, “A General and unspecified” and “R Respiratory” [28]. However, knowledge is lacking on how older people utilise the OOH services in Norway.

The aim of this study was to investigate the contact characteristics among people 70 years and older including time and mode of contact, RFE, priority degree, and initial response, as well as identifying factors associated with urgent priority degree.

## Methods

### Design and setting

This cross-sectional study was based on data from the “Watchtower project” during the period 1 January 2014 until 31 December 2017. The Watchtower project is a sentinel network of representative OOH emergency primary health care centres in Norway and has been collecting data on their activity since 2007. During the study period, the sentinel network consisted of seven OOH-districts covering 18 municipalities. The total number of inhabitants from the OOH-districts ranged from 234,480 in 2014 to 240,890 in 2017. The average number of inhabitants corresponded to approximately 4.6% of the total Norwegian population during the study period. The Watchtower project is funded by the National Centre for Emergency Primary Health Care, and further project details are described thoroughly elsewhere [29, 30].

During the four-year study period, 255,508 medical contacts were registered among all age groups, an average of 63,877 contacts per year, corresponding to 269 contacts/1000 inhabitants per year. Our study only included contacts from patients 70 years and older. The total number of inhabitants 70 years and older living in the OOH-districts ranged from 23,353 in 2014 to 26,396 in 2017, a yearly average of 24,751 inhabitants.

### Variables

When the patient (or someone on behalf of the patient) contacted the OOH service, the OOH nurse registered the following: sex, age, day of week, time of day, mode of contact, RFE, priority degree, and first response initiated.

*- Time of day*: day (8 a.m. to 3.29 p.m.), evening (3.30 p.m. 10.59 p.m.) and night (11 p.m. to 7.59 a.m.). Due to the organizational model of emergency primary health care services in Norway, we excluded all daytime contacts Monday-Friday (except holidays) because during this time, the RGPs’ facilities are open, and they are responsible for the patients who seek emergency medical health care.

*Mode of contact:* was categorized as: 1) Telephone call from patient, next of kin or caregiver, 2) Direct attendance, 3) Telephone call from health professionals, 4) Emergency Medical Communication Centre (EMCC) or alarm or 5) Other (police, social service etc.).

*RFE:* was registered using the International Classification of Primary Care, second edition (ICPC-2). The RFE was determined from the patient’s main complaint or stated reason for contact. ICPC-2 is accepted as a mode of classifying RFEs in primary health care and has proven to be a reliable tool [31, 32].

*Priority degree:* Each contact was assigned a colour-coded priority degree: green “non-urgent”, yellow “urgent” and red “acute” in accordance with the Norwegian Index of Emergency Medical Assistance (Norwegian Index); this is a decision support tool used to determine response patterns, degree of urgency, and to provide emergency medical instruction guidance in lifesaving first aid [33].

*First response initiated:* was categorized as: 1) Telephone advice only, by nurse, 2) Telephone advice only, by doctor, 3) Medical consultation with a doctor, 4) Medical consultation with a nurse, 5) Call out by ambulance and doctor, 6) Home visit by doctor or 7) Other (call out by ambulance (no doctor), referral to police/RGP).

### Statistical methods

Frequencies, percentages and prevalence rates (medical contacts per 1000 inhabitants per year) were calculated for all variables. Relative risks (RR) were calculated to describe sex and age trends within the variables. To investigate variables associated with urgent priority degree, we performed a log-binomial regression analysis in a two-step manner. The dependent variable was degree of urgency, dichotomized into non-urgent (green) and urgent/acute (yellow/red). The independent variables included were sex, time of day, weekdays (Monday 8 a.m. to Friday 3.29 p.m.) vs. weekend (Friday 3.30 p.m. to Monday 7.59 a.m.), the different modes of contact (telephone from patient, next of kin, caregiver, telephone from health professionals and direct attendance) and RFE by ICPC-2 chapter. RR, 95% confidence interval (CI) and *p*-values were estimated. To account for possible dependence within each OOH-district, we used robust standard error estimates. First, each independent variable listed above was tested in an unadjusted model; second, the variables considered significant were applied together in an adjusted model. In the adjusted model, we used iterated, reweighted least-squares optimization of the deviance. Due to problems with convergence, we could not include the variable time of day in the adjusted model. The reported RRs, CIs and *p*-values for this variable were adjusted for a modified RFE-variable in addition to the other variables (see Table 4).

In our data material, 9.9% of the cases had missing ICPC-2 codes (Table 1, RFE unknown). For the other variables, missing data were insignificant. No imputation methods for missing values were used.

**Table 1:** OOH contact characteristics of patients 70 years and older (*n* = 38,293)

Contact characteristics	n	(%)	Rate^a^
Age groups			
70–74 years	9353	(24.4)	253
75–79 years	8253	(21.6)	328
80–84 years	8113	(21.2)	458
85–89 years	7088	(18.5)	594
≥90 years	5486	(14.3)	769
Sex			
Women	22,140	(57.8)	401
Men	16,146	(42.2)	369
Sex unknown	7	(0.0)	0
Time of day			
Daytime (08:00–15:29) ^b^	12,089	(31.6)	122
Evening (15:30–22:59)	20,025	(52.3)	202
Night (23:00–07:59)	6179	(16.1)	62
Day of week			
Monday	3989	(10.4)	40
Tuesday	3527	(9.2)	36
Wednesday	3641	(9.5)	37
Thursday	3831	(10.0)	39
Friday	4248	(11.1)	43
Saturday	10,214	(26.7)	103
Sunday	8843	(23.1)	89
Mode of contact			
Telephone from patient, next of kin, caregiver	19,896	(52.0)	201
Telephone from health professionals	12,483	(32.8)	126
Direct attendance	3198	(8.4)	32
EMCC ^c^/alarm	2513	(6.6)	25
Other (police, social service)	198	(0.5)	2
Mode of contact unknown	5	(0.0)	0
Reason for encounter (ICPC-2 chapter)			
General and unspecified (A)	8109	(21.2)	82
Musculoskeletal (L)	5189	(13.6)	52
Respiratory (R)	4937	(12.9)	50
Urological (U)	3557	(9.3)	36
Digestive (D)	3354	(8.8)	34
Cardiovascular (K)	2436	(6.4)	25
Skin (S)	2172	(5.7)	22
Neurological (N)	1526	(4.0)	15
Psychological (P)	1218	(3.2)	12
Eye (F)	714	(1.9)	7
Endocrine/metabolic/nutrition (T)	480	(1.3)	5
Other chapters ^d^	797	(2.1)	8
Reason for encounter unknown	3804	(9.9)	39
Priority degree			
Green, not urgent	23,110	(60.4)	233
Yellow, urgent	12,574	(32.8)	127
Red, acute	2554	(6.7)	26
Priority degree unknown	55	(0.1)	0
Initial response			
Medical consultation by doctor	18,530	(48.4)	187
Telephone advice only, by doctor	6764	(17.7)	68
Telephone advice only, by nurse	6546	(17.1)	66
Home visit by doctor	2296	(6.0)	23
Call out by ambulance and doctor	1819	(4.8)	18
Other (call out by ambulance (no doctor)/ref. to police)	1790	(4.7)	18
Medical consultation by nurse	450	(1.2)	5
Initial response unknown	98	(0.6)	1

^a^ Rate per 1000 inhabitants per year; ^b^ Saturdays, Sundays, Holidays; ^c^ EMCC: Emergency Medical Communication Centre; ^d^ Ear (H), Blood (B), Male genital (Y), Female genital (X), Social problems (Z) Pregnancy and family planning (W)

IBM SPSS Statistics 25 and StataSE16 were used to analyse the data, and the significance level was set to α = 0.05.

### Ethics

This study is part of the Watchtower project and has been approved by the Norwegian Centre for Research Data (SAK 31590). All data are anonymized, no patient identifiable data were recorded at any time, hence there was no need for informed consent from participants. The Regional Committee for Medical and Health Research Ethics did not consider it necessary to assess the study for approval (2012/1094/REC West).

## Results

### Inhabitants and contact characteristics

During the four-year study period, a total of 38,293 contacts from patients 70 years and older were registered, an average of 9573 contacts each year (387 contacts/1000 inhabitants 70 years and older per year). The absolute number of contacts decreased with higher age, but the rate was three times higher in the oldest age group (≥90 years) compared to the youngest age group (70–74 years) (769 vs. 253 contacts/1000 inhabitants per year) (Table 1).

The mean age was 81 years (range 70 to 107 years), and women had the highest contact rates compared with men (401 vs. 369). Saturday was the day with highest percentage of contacts (26.7%), followed by Sunday (23.1%). Additionally, we found that Monday (10.4%) and Friday (11.1%) had a slightly higher number of contacts than the remaining weekdays (9.2–10.0%). Over half (52.0%) of the contacts came from patients, next of kin or caregiver by telephone while one-third (32.8%) of the contacts were telephone from health professionals (Table 1). Contacts by telephone from health professionals were seven times more common among patients 85 years and older compared to patients between 70 and 74 years (see Additional file 2). The proportion of direct attendance was low (8.4%) compared to the telephone contacts (Table 1), and it decreased even further with higher age (see Additional file 2). The majority of the contacts (60.4%) were set to green (not urgent) priority degree, followed by yellow (urgent) (32.9%) and red (acute) (6.7%) (Table 1). We also found a higher rate among women compared with men in contacts resulting in green priority degree (251 vs. 211) (see Additional file 1).

### RFE by ICPC-2 chapter and code

The most frequent RFE within the ICPC-2 chapters was “A General and unspecified” (21.2%) followed by chapter “L Musculoskeletal” (13.6%) and R “Respiratory” (12.9%). The chapters “U Urological” and “D Digestive” were also relatively common RFEs (Table 1). “A General and unspecified” was the ICPC-2 chapter that increased the most with higher age (compared with chapter “L” and “R”) (see Additional file 1). The relative risk (RR) of contacting the OOH service with an “A General and unspecified” RFE was 5.2 (men) and 4.4 (women) times higher in persons 90 years and older compared with persons between 70 and 74 years of age. Women with contacts from the “L Musculoskeletal” chapter had a higher rate compared with men (60 vs. 42) (see Additional file 1).

We registered 493 different ICPC-2 codes (RFEs). Table 2 presents the 20 most frequent RFEs, which added up to 46.3% of all the contacts. The most common RFE were “R02 Shortness of breath/dyspnoea” (5.0%), followed by “D01 Abdominal pain/cramps general” (3.8%).

**Table 2:** The most frequent RFEs by ICPC-2 codes (*n* = 34,489)

ICPC-2 code	ICPC-2 code name	n	(%)	Rate ^a^
R02	Shortness of breath/dyspnoea	1728	(5.0)	17
D01	Abdominal pain/cramps general	1318	(3.8)	13
U71	Cystitis/urinary infection other	1189	(3.4)	12
A13	Concern about/fear of medical treatment	1095	(3.2)	11
A11	Chest pain	1001	(2.9)	10
A03	Fever	930	(2.7)	9
A29	General symptom/complaint other	828	(2.4)	8
S18	Laceration/cut	806	(2.3)	8
A28	Limited function/disability	745	(2.2)	7
U29	Urinary symptom/complaint other	717	(2.1)	7
L02	Back symptom/complaint	651	(1.9)	7
R05	Cough	628	(1.8)	6
L13	Hip symptom/complaint	621	(1.8)	6
A96	Death	604	(1.8)	6
N17	Vertigo/dizziness	581	(1.7)	6
R81	Pneumonia	551	(1.6)	6
L17	Foot/toe symptom/complaint	538	(1.6)	5
L14	Leg/thigh symptom/complaint	494	(1.4)	5
R29	Respiratory symptom/complaint	478	(1.4)	5
A97	No disease	469	(1.4)	5
	Other RFEs	18,517	(53.7)	187

^a^ Rate per 1000 inhabitants 70 years and older per year

### Most frequent RFE and first initial response

Almost half (48.4%) of the contacts resulted in a medical consultation with a doctor. The proportion of contacts that resulted in telephone advice from a nurse and doctor were about the same, while home visits by a doctor were infrequent (6.0%) (Table 1). We also found a higher rate among women compared to men regarding telephone advice from a nurse and doctor (147 vs. 119) (see Additional file 2). When the first response initiated was a home visit by a doctor, the RR was almost 14 times higher in patients 90 years and older compared to patients between 70 and 74 years in both men and women (see Additional file 2).

Table 3 shows the most common RFEs by first initial response. The most common RFEs that led to a medical consultation with a doctor were “R02 Shortness of breath” (5.3%) and “D01 Abdominal pain/cramps general” (4.7%). “A13 Concerns about/fear of medical treatment” (8.0%) was the RFE that received telephone advice from a nurse or doctor most frequently. In home visits by a doctor, the RFE code “A96 Death” (13.3%) had almost twice as many contacts compared to “R02 Shortness of breath/dyspnoea” (7.6%).

**Table 3:** The most frequent RFEs by ICPC-2 codes and initial response; medical consultation doctor, telephone advice, home visit by doctor, call out by ambulance and doctor (*n* = 32,386)

ICPC-2 code	Initiated response & ICPC-2 code name	n	(%)	Rate ^a^
*Medical consultation doctor (n = 16,862)*				
R02	Shortness of breath/dyspnoea	898	(5.3)	9
D01	Abdominal pain/cramps general	786	(4.7)	8
S18	Laceration/cut	627	(3.7)	6
U71	Cystitis/urinary infection	604	(3.6)	6
A11	Chest pain	491	(2.9)	5
A03	Fever	440	(2.6)	4
	Other	13,016	(77.2)	131
*Telephone advice only (n = 11,856)*				
A13	Concern about/fear of medical treatment	948	(8.0)	10
A28	Limited function/disability	581	(4.9)	6
U71	Cystitis/urinary infection	495	(4.2)	5
A29	General symptom/complaint	376	(3.2)	4
R02	Shortness of breath/dyspnoea	367	(3.1)	4
D01	Abdominal pain/cramps general	358	(3.0)	4
	Other	8731	(73.6)	88
*Home visit by doctor (n = 2043)*				
A96	Death	274	(13.4)	3
R02	Shortness of breath/dyspnoea	155	(7.6)	1
R81	Pneumonia	121	(5.9)	1
A03	Fever	106	(5.5)	1
A29	General symptom/complaint other	66	(3.2)	1
D01	Abdominal pain/cramps general	56	(2.7)	1
	Other	1265	(61.7)	13
*Call out by ambulance and doctor (n = 1625)*				
A11	Chest pain NOS	252	(15.0)	3
R02	Shortness of breath/dyspnoea	159	(9.8)	2
K01	Heart pain	149	(9.2)	2
K90	Stroke/cerebrovascular accident	108	(6.7)	1
D01	Abdominal pain/cramps general	59	(3.7)	1
L13	Hip symptom/complaint	51	(3.2)	1
	Other	847	(52.4)	9

^a^ Rate per 1000 inhabitants 70 years and older per year

### Variables associated with urgent priority degree

The results from the log-binomial regression model are presented in Table 4. The unadjusted model found all variables to be significantly associated with urgent priority degree (*p* < 0.001).

**Table 4:** Unadjusted and adjusted log-binomial regression model for urgent priority by; sex, age, time of day, week, mode of contact and RFE by ICPC-2 chapter

Variables	n	(%)	Unadjusted	*p* value	Adjusted	*p* value
			RR (95% CI)		RR (95% CI)	
Sex						
Women	8248	(37.3)	Ref.		Ref.	
Men	6875	(42.7)	1.14 (1.11–1.18)	**< 0.001**	1.13 (1.09–1.17)	**< 0.001**
Age groups						
70–74	3586	(38.4)	Ref.		Ref.	
75–79	3285	(39.9)	1.04 (1.01–1.07)	**0.018**	1.05 (1.01–1.8)	**0.010**
80–84	3143	(38.8)	1.01 (0.96–1.06)	0.698	0.99 (0.93–1.05)	0.718
85–89	2917	(41.2)	1.07 (1.03–1.12)	**0.001**	1.08 (1.02–1.13)	**0.007**
90+	2197	(40.1)	1.04 (1.01–1.08)	**0.011**	1.06 (1.01–1.10)	**0.012**
Time of day						
Day 08:00–15:29	4019	(33.3)	0.83 (0.80–0.87)	**< 0.001**	0.86 (0.82–0.90)	< 0.001*
Evening 15:30–22:59	8044	(40.2)	Ref.		Ref.	
Night 23:00–07:59	3265	(49.7)	1.24 (1.12–1.37)	**< 0.001**	1.22 (1.12–1.32)	< 0.001*
Week						
Weekday	6602	(43.2)	1.16 (1.08–1.26)	**< 0.001**	1.13 (1.06–1.20)	**< 0.001**
Weekend	8526	(37.1)	Ref.		Ref.	
Mode of contact						
Telephone patient, next of kin, caregiver	6772	(34.1)	Ref.		Ref.	
Direct attendance	1226	(38.4)	1.13 (1.05–1.21)	**0.001**	1.13 (1.04–1.22)	**0.003**
Telephone health professionals	5173	(41.5)	1.22 (1.12–1.32)	**< 0.001**	1.21 (1.12–1.31)	**< 0.001**
RFE by ICPC-2 chapter						
(A) General and unspecified	3064	(37.9)	Ref.		Ref.	
(L) Musculoskeletal	1992	(38.4)	1.01 (0.93–1.10)	0.754	1.13 (1.02–1.25)	**0.015**
(R) Respiratory	2373	(48.1)	1.27 (1.19–1.35)	**< 0.001**	1.40 (1.30–1.51)	**< 0.001**
(U) Urological	1028	(28.9)	0.76 (0.61–0.96)	**0.022**	0.86 (0.68–1.10)	0.235
(D) Digestive	1400	(41.8)	1.10 (1.02–1.19)	**0.009**	1.22 (1.10–1.34)	**< 0.001**
(K) Cardiovascular	1616	(66.4)	1.75 (1.63–1.89)	**< 0.001**	1.85 (1.74–1.96)	**< 0.001**
(S) Skin	672	(31.0)	0.82 (0.74–0.91)	**< 0.001**	0.91 (1.81–1.02)	0.091
(N) Neurological	827	(54.3)	1.43 (1.26–1.63)	**< 0.001**	1.55 (1.36–1.77)	**< 0.001**
(P) Psychological	341	(28.0)	0.74 (0.67–0.81)	**< 0.001**	0.78 (0.71–0.86)	**< 0.001**
(F) Eye	170	(23.8)	0.63 (0.56–0.71)	**< 0.001**	0.75 (0.66–0.85)	**< 0.001**
(T) Endocrine/metabolic/nutrition	164	(34.2)	0.90 (0.71–1.15)	0.416	0.93 (0.75–1.15)	0.507
Other chapters ^a^	231	(29.1)	0.77 (0.68–0.87)	**< 0.001**	0.85 (0.78–0.94)	**0.001**

Log-binominal regression analyses using women, age group 70–74, evening 15:30–22:59, weekend, telephone from patient or next of kin or caregiver and ICPC-2 chapter “A General and unspecified” as reference; We used clustered standard errors to adjust for possible dependence in the seven OOH-districts; Significant values are marked as bold; ^a^Ear (H), Blood (B), Male genital (Y), Female genital (X), Social problems (Z) Pregnancy and family planning (W).*Due to model limitations with too many variables with multiple categories in the adjusted model, the variable “Time of day” was excluded from the adjusted analysis. The reported RR, CI and p-values for this variable were adjusted for a modified RFE-variable (with the categories “A General and unspecified”, “L Musculoskeletal”, “R Respiratory” and “Other chapters”) in addition to the other variables

In the adjusted model, the RFE chapters “K Cardiovascular”, “N Neurological”, “R Respiratory” and “D Digestive” were strongest associated with urgent priority degree (all *p* < 0.001). The RR for urgent priority degree was 85% higher when the patient had a contact within chapter “K Cardiovascular” compared to “A General and unspecified”. The RRs for the chapters “N Neurological”, “R Respiratory” and “D Digestive” were 55, 40 and 22%, respectively. The RR was 13% higher when the patients attended the OOH centre directly (*p* = 0.003) and 21% higher when a health professional called the OOH centre (*p* < 0.001) (compared with telephone from patient, next to kin or caregiver). The RR for receiving an urgent priority degree was 13% higher when both men (*p* < 0.001) contacted the OOH service (compared with women) and when the contact happened on a weekday (compared to weekends). Lastly, within the age groups 75–79 years (*p* = 0.010), 85–89 years (*p* = 0.007) and 90 years and older (*p* = 0.012) the RRs for receiving an urgent priority were 5–8% higher (compared with 70–74 years); hence we found no particular increase in urgent priority degree with higher age.

## Discussion

The contact rate increased with higher age. Telephone contact from health professionals calling on behalf of the patient was found to be a common mode of contact, especially for those 85 years and older. In addition, the rate of direct attendance was low. The most common RFE by chapter was “A General and unspecified”, and the rate increased with higher age. Home visits by a doctor were few, but the rate increased considerably for patients 85 years of age or older. Lastly, variables significantly associated with urgent priority degree were RFEs regarding cardiovascular, neurological, respiratory and digestive issues in addition to telephone calls from health professionals, direct attendance, contacts on weekdays and contacts from men.

The overall contact rate in our study population was 387/1000 inhabitants, much higher than the overall rate for the whole population (269/1000). The rate increased significantly with higher age. Similar trends were found in studies investigating telephone contacts from older people at the OOH service in England and Scotland [5], and visits to the emergency department in Chicago [4]. The increased utilization of OOH services could be explained by higher age itself, but is more likely due to factors such as frailty and multimorbidity [34, 35].

The majority of contacts came by telephone and almost one–third were calls from health professionals. This illustrates that older people contact the OOH services indirectly, and that the OOH nurse frequently communicates with a person calling on behalf of the patient. A recently published qualitative study from Ireland reported that older people find it difficult to contact the OOH services when they become ill. Concerns about transportation and having to ask family members or neighbours for help, especially at night, were associated with a reluctance to seek help [20]. Older persons may therefore find it less problematic to contact the OOH service by telephone instead of going in person.

We found a high number (493) of different RFEs. A Danish study found 392 different RFEs among telephone contacts in all age groups [36]. The large number in our study shows that older people have broadly varied symptoms, conditions, and issues. Furthermore, the most common RFE by chapter was “A General and unspecified”, and the proportion increased with higher age. A similar result was found in an Australian study [37], but the results were from general practice and not an OOH setting, which may affect the sample of patients and RFEs. Chapter “A General and unspecified” has previously proven to be common among all age groups [28]. However, as the rate of this RFE increased among older people, one might question if this age group is more difficult to interpret due to diffuse symptoms and complaints. We therefore specifically investigated some single RFEs within the chapter “A General and unspecified”.

The most common RFE code within the chapter “A General and unspecified” was “A13 Concern about/fear of medical treatment”. Our study material cannot substantiate what these 8% of the contacts involved, but it may be related to polypharmacy or lack of health literacy. Data from the Norwegian Prescription Database showed that more than half of all registered women and men 65 to 74 years old had five or more prescriptions in 2016 and the proportion increased considerably with higher age [38]. Moreover, studies have shown that health literacy decreases with aging [39], and that physicians tend to overestimate patients’ literacy level [40]. This underscores that physicians need to be aware of the patient’s literacy level, and target communication accordingly, especially when it comes to new prescriptions or changes in medication.

The ICPC-2 codes “A28 Limited function/disability” and “A29 General symptom/complaint” were also common RFEs in our study. Combined, these codes accounted for the highest proportion of telephone advice responses. Several studies have reported a moderate to high frequency of atypical presentation in acute illness among older people presenting to emergency departments [11–19]. “A28 Limited function/disability” and “A29 General symptom/complaint” could possibly fit such a term. Previous studies have included terms like “decreased general condition” [13], “non-specific complaints” [11, 18] or “general weakness” [19] in atypical presentation. Several studies have found that such diffuse and non-specific symptoms in many cases represent underlying serious illness, like stroke, pneumonia, or ischemic heart disease [12, 14, 18, 19].

Contacts resulting in home visits by doctors were few in our study, although the rate increased with higher age. Other studies have also found an increase in home visits among older people [5, 7]. In the past three decades, there has been a steady decrease in the number of home visits [41]. Simultaneously, there has been a trend towards fewer and larger OOH districts. The smallest OOH centres are those that perform home visits most often [3, 41], and long distances to the OOH centre affects Norwegian inhabitants’ utilization of the OOH centres [42]. Therefore, the municipalities must facilitate for home visits among the older people when organizing their service. By doing so, the frailest patients for whom it is undesirable and potentially harmful to travel to the OOH centres, can receive the help they need.

Direct attendance was associated with urgent priority degree. This fits well with findings from a qualitative study investigating nurse practitioners triage decisions. The study found that the patient’s physical appearance and “how stable and unstable they look” were cues that strongly affected their triage decision [43].

Contacts from men were significantly associated with urgent priority degree, in line with a study from the Netherlands [44]. A possible explanation for our finding may be that OOH-nurses generally perceive men as more urgent cases than women. A study of triage decisions found that nurses were more likely to consider the situation as significant when men presented with complaints suggesting acute myocardial infarction [43]. The gender differences in priority degree may also be explained by gender differences in symptom presentation. It has previously been reported that women tend to present more often with atypical symptoms [45] which may result in under-triage [14]. However, the gender effect on symptom presentation is debatable, and authors have reported that symptom presentation is affected by other factors [45], and not by gender itself. Our data does not contain information about the presentation form, only one ICPC-2 code per contact. Therefore, future studies should include symptom presentation when investigating the OOH nurses’ triage decisions to elaborate the gender differences among older people.

An educational consequence of our findings may be that specific training of OOH nurses in the variety of symptoms and presentations of older people should be strengthened. In addition, there could be more incorporation of older persons’ characteristics in decision support tools. It would also be of benefit for the OOH nurses if simple screening tools were available to detect older frail patients by telephone, which unfortunately is not the case at the OOH emergency primary health care services in Norway at the present time.

### Strengths and limitations

To our knowledge, studies of OOH contact characteristics and factors associated with the degree of urgency among the older population in Norway have not been conducted previously. Our study also has a high number of registrations. We consider 9% missing ICPC-2 codes as a low percentage. One possible reason for lacking data might be difficulties in deciding the RFE among older people, but a previous study that analysed the same material found that there were no age differences in lacking ICPC-2 codes [30]. We used both adjusted and unadjusted models in the log binomial regression analyses in order to take into account the effects of other factors that may have been missed in an unadjusted model alone.

Our data sample has been collected from the “Watchtower project” which was initiated in 2006. The project was designed to be representative of the OOH services in Norway by considering the population size, degree of population change, age and sex composition, degree of centrality, type of business in the municipalities, municipal economy and income level. Representativeness has been validated previously [25]. Nevertheless, we cannot exclude the possibility that changes in the seven OOH districts in the subsequent years may have resulted in a lower degree of representativity.

Our data do not include variables about frailty, multimorbidity, functional level or socioeconomic aspects. We registered contacts, not patients. We therefore do not know how many times the same patient has contacted the OOH service during the study period. The nurses only register one symptom per patient, and this may be problematic given that older people often have multiple symptoms and a possibly complex disease composition.

## Conclusions

This study provides important information about the Norwegian older inhabitants’ contact with the OOH emergency primary health care services. There are a wide variety of RFEs, and the contact rate is high and increases with higher age. Telephone contact is most common. The OOH staff frequently identify older people as having “general and unspecified” reasons for encounters. OOH nursing staff would benefit from having screening tools and enhanced geriatric training to best support this vulnerable group when these individuals call the OOH service.

## Supplementary information

**Additional file 1.** Age and sex differences within RFE by ICPC-2 chapter, time of day and priority degree.

**Additional file 2.** Age and sex differences within mode of contact and first response initiated.

## Data Availability

Due to the approvals from the Regional committee for Medical and Health Research Ethics and the Privacy ombudsman for research, the data are not currently available, but are available from the corresponding author on reasoned/justified request.

## Abbreviations

EMCC: Emergency Medical Communication Centre
ICPC-2: International Classification of Primary Care 2nd edition
ICD-10: International Classification of Diseases 10th revision
OOH: Out-of-hours
RFE: Reason for encounter
RGP: Regular general practitioner
RR: Relative risk
