# Supplementary material for: Contact characteristics and factors associated with the degree of urgency among older people in emergency primary health care: a cross-sectional study
Source: BMC Health Serv Res. 2020 Apr 22;20:345. doi: 10.1186/s12913-020-05219-0 (PMC7178956; doi:10.1186/s12913-020-05219-0)
Supplement: Supplementary file 1 — Additional file 1. Age and sex differences within RFE by ICPC-2 chapter, time of day and priority degree. [file 12913_2020_5219_MOESM1_ESM.docx]

| RFE by ICPC-2 chapter | (A) General and unspecified (*n* = 8109) | | | |  | (L) Musculoskeletal (*n* = 5188) | | | |  | (R) Respiratory (*n* = 4937) | | | |
| --- | --- | --- | --- | --- | --- | --- | --- | --- | --- | --- | --- | --- | --- | --- |
|  | n | (%) | Rate ^1^ | RR^2^ |  | n | (%) | Rate | RR |  | n | (%) | Rate | RR |
| Men (*n* = 7676) |  |  | 81 |  |  |  |  | 42 |  |  |  |  | 51 |  |
| 70-74 years | 820 | (41.0) | 45 | Ref. |  | 594 | (29.7) | 32 | Ref. |  | 587 | (29.3) | 32 | Ref. |
| 75-79 years | 785 | (43.9) | 67 | 1.5 |  | 462 | (25.8) | 39 | 1.2 |  | 541 | (30.3) | 46 | 1.4 |
| 80-84 years | 845 | (49.9) | 112 | 2.4 |  | 372 | (21.9) | 49 | 1.5 |  | 478 | (28.2) | 63 | 2.0 |
| 85-89 years | 677 | (50.5) | 159 | 3.5 |  | 302 | (22.5) | 72 | 2.2 |  | 361 | (26.9) | 86 | 2.7 |
| ≥90 years | 446 | (52.3) | 232 | 5.2 |  | 154 | (18.1) | 80 | 2.5 |  | 252 | (29.6) | 131 | 4.1 |
|  |  |  |  |  |  |  |  |  |  |  |  |  |  |  |
| Women (*n* = 10,558) |  |  | 82 |  |  |  |  | 60 |  |  |  |  | 49 |  |
| 70-74 years | 804 | (36.2) | 43 | Ref. |  | 752 | (33.8) | 40 | Ref. |  | 667 | (30.0) | 36 | Ref. |
| 75-79 years | 817 | (39.7) | 61 | 1.4 |  | 725 | (35.2) | 54 | 1.3 |  | 518 | (25.1) | 39 | 1.1 |
| 80-84 years | 897 | (42.6) | 88 | 2.0 |  | 635 | (30.1) | 63 | 1.6 |  | 576 | (27.3) | 57 | 1.6 |
| 85-89 years | 1023 | (46.7) | 132 | 3.1 |  | 647 | (29.5) | 84 | 2.1 |  | 520 | (23.7) | 67 | 1.9 |
| ≥90 years | 995 | (50.3) | 191 | 4.4 |  | 545 | (27.6) | 105 | 2.6 |  | 437 | (22.1) | 89 | 2.3 |
|  |  |  |  |  |  |  |  |  |  |  |  |  |  |  |
| Time of day | Day (08:00-15:29) (*n* = 12,086) | | | |  | Evening (15:30-22:59) (*n* = 20,023) | | | |  | Night (23:00-07:59) (*n* = 6177) | | | |
| Men (*n* = 16,146) |  |  | 112 |  |  |  |  | 194 |  |  |  |  | 63 |  |
| 70-74 years | 1276 | (29.2) | 69 | Ref. |  | 2230 | (53.4) | 127 | Ref. |  | 759 | (17.4) | 41 | Ref. |
| 75-79 years | 1167 | (30.0) | 99 | 1.4 |  | 2041 | (52.4) | 174 | 1.3 |  | 684 | (17.6) | 58 | 1.4 |
| 80-84 years | 1039 | (29.4) | 137 | 2.0 |  | 1882 | (53.2) | 249 | 2.0 |  | 616 | (17.4) | 81 | 2.0 |
| 85-89 years | 867 | (32.3) | 206 | 3.0 |  | 1372 | (51.1) | 372 | 2.9 |  | 447 | (16.6) | 107 | 2.6 |
| ≥90 years | 555 | (33.3) | 288 | 4.2 |  | 875 | (52.5) | 455 | 3.6 |  | 236 | (14.2) | 123 | 3.0 |
|  |  |  |  |  |  |  |  |  |  |  |  |  |  |  |
| Women (*n* = 22,140) |  |  | 130 |  |  |  |  | 209 |  |  |  |  | 62 |  |
| 70-74 years | 1584 | (31.8) | 85 | Ref. |  | 2607 | (52.3) | 140 | Ref. |  | 797 | (16.0) | 43 | Ref. |
| 75-79 years | 1393 | (32.0) | 104 | 1.2 |  | 2248 | (51.6) | 167 | 1.2 |  | 717 | (16.5) | 53 | 1.2 |
| 80-84 years | 1434 | (31.3) | 141 | 1.7 |  | 2418 | (52.9) | 238 | 1.7 |  | 723 | (15.8) | 71 | 1.7 |
| 85-89 years | 1517 | (34.5) | 196 | 2.3 |  | 2238 | (50.9) | 289 | 2.1 |  | 646 | (14.7) | 83 | 1.9 |
| ≥90 years | 1254 | (32.8) | 241 | 2.8 |  | 2012 | (52.7) | 386 | 2.8 |  | 552 | (14.5) | 106 | 2.5 |
|  |  |  |  |  |  |  |  |  |  |  |  |  |  |  |
| Priority degree | Green (*n* =23,108) | | | |  | Yellow (*n* = 12,570) | | | |  | Red (*n* = 2553) | | | |
| Men (*n* = 16,116) |  |  | 211 |  |  |  |  | 131 |  |  |  |  | 26 |  |
| 70-74 years | 2522 | (57.9) | 137 | Ref. |  | 1540 | (35.3) | 84 | Ref. |  | 295 | (6.8) | 16 | Ref. |
| 75-79 years | 2234 | (57.5) | 190 | 1.4 |  | 1363 | (35.1) | 116 | 1.4 |  | 288 | (7.4) | 25 | 1.6 |
| 80-84 years | 2021 | (57.2) | 267 | 1.9 |  | 1242 | (35.2) | 164 | 2.0 |  | 269 | (7.6) | 36 | 2.4 |
| 85-89 years | 1526 | (57.0) | 364 | 2.7 |  | 966 | (36.1) | 230 | 2.7 |  | 186 | (6.9) | 44 | 2.8 |
| ≥90 years | 938 | (56.4) | 488 | 3.6 |  | 612 | (36.8) | 318 | 3.8 |  | 144 | (6.9) | 59 | 3.7 |
|  |  |  |  |  |  |  |  |  |  |  |  |  |  |  |
| Women (*n* = 22,115) |  |  | 254 |  |  |  |  | 124 |  |  |  |  | 25 |  |
| 70-74 years | 3231 | (64.9) | 173 | Ref. |  | 1462 | (29.3) | 78 | Ref. |  | 289 | (5.8) | 15 | Ref. |
| 75-79 years | 2721 | (62.5) | 203 | 1.2 |  | 1370 | (31.5) | 102 | 1.3 |  | 263 | (6.0) | 20 | 1.3 |
| 80-84 years | 2938 | (64.3) | 289 | 1.7 |  | 1337 | (29.3) | 132 | 1.7 |  | 294 | (6.4) | 29 | 1.9 |
| 85-89 years | 2632 | (59.9) | 340 | 2.0 |  | 1456 | (33.1) | 188 | 2.4 |  | 308 | (7.0) | 40 | 2.6 |
| ≥90 years | 2345 | (61.5) | 450 | 2.6 |  | 1222 | (32.0) | 235 | 3.0 |  | 247 | (6.5) | 47 | 3.1 |

**Additional file 1** Age and sex differences within RFE by ICPC-2 chapter, time of day and priority degree.

^1^Rate per 1000 inhabitants 70 years and older per year; ^2^Relative risk
