# Supplementary material for: Contact characteristics and factors associated with the degree of urgency among older people in emergency primary health care: a cross-sectional study
Source: BMC Health Serv Res. 2020 Apr 22;20:345. doi: 10.1186/s12913-020-05219-0 (PMC7178956; doi:10.1186/s12913-020-05219-0)
Supplement: Supplementary file 2 — Additional file 2. Age and sex differences within mode of contact and first response initiated. [file 12913_2020_5219_MOESM2_ESM.docx]

| First response initiated | Medical examination by doctor  (*n* = 18,529) | | | |  | Telephone advice by doctor or nurse  (*n* = 13,307) | | | |  | Acute response ambulance and doctor  (*n* = 1819) | | | |  | Home visits by a doctor (*n* = 2295) | | | |
| --- | --- | --- | --- | --- | --- | --- | --- | --- | --- | --- | --- | --- | --- | --- | --- | --- | --- | --- | --- |
|  | n | (%) | Rate ^1^ | RR^2^ |  | n | (%) | Rate | RR |  | n | (%) | Rate | RR |  | n | (%) | Rate | RR |
| Men (*n* = 15,189) |  |  | 186 |  |  |  |  | 119 |  |  |  |  | 19 |  |  |  |  | 23 |  |
| 70-74 years | 2583 | 62.5 | 141 | Ref. |  | 1182 | 28.7 | 64 | Ref. |  | 220 | 5.3 | 12 | Ref. |  | 147 | 3.6 | 8 | Ref. |
| 75-79 years | 2068 | 56.6 | 176 | 1.2 |  | 1230 | 33.7 | 105 | 1.6 |  | 183 | 5.0 | 16 | 1.3 |  | 174 | 4.8 | 15 | 1.9 |
| 80-84 years | 1708 | 51.6 | 226 | 1.6 |  | 1164 | 35.2 | 154 | 2.4 |  | 197 | 6.0 | 26 | 2.2 |  | 239 | 7.3 | 32 | 4.0 |
| 85-89 years | 1145 | 45.4 | 273 | 1.9 |  | 993 | 39.4 | 238 | 3.7 |  | 141 | 5.6 | 34 | 2.8 |  | 242 | 9.7 | 58 | 7.3 |
| ≥90 years | 623 | 40.0 | 324 | 2.3 |  | 631 | 40.5 | 328 | 5.0 |  | 94 | 6.0 | 49 | 4.1 |  | 211 | 13.5 | 110 | 13.8 |
|  |  |  |  |  |  |  |  |  |  |  |  |  |  |  |  |  |  |  |  |
| Women (*n* = 20,761) |  |  | 188 |  |  |  |  | 147 |  |  |  |  | 18 |  |  |  |  | 23 |  |
| 70-74 years | 2888 | 61.5 | 155 | Ref. |  | 1514 | 32.2 | 81 | Ref. |  | 175 | 3.7 | 9 | Ref. |  | 121 | 2.6 | 6 | Ref. |
| 75-79 years | 2328 | 56.6 | 173 | 1.1 |  | 1444 | 35.1 | 107 | 1.3 |  | 166 | 4.0 | 12 | 1.3 |  | 172 | 4.2 | 13 | 2.2 |
| 80-84 years | 2105 | 48.4 | 207 | 1.3 |  | 1749 | 40.4 | 172 | 2.1 |  | 235 | 5.4 | 23 | 2.5 |  | 236 | 5.5 | 23 | 3.8 |
| 85-89 years | 1853 | 45.3 | 239 | 1.5 |  | 1686 | 41.2 | 218 | 2.7 |  | 227 | 5.5 | 29 | 3.2 |  | 329 | 8.0 | 43 | 7.2 |
| ≥90 years | 1226 | 34.7 | 235 | 1.5 |  | 1705 | 48.3 | 327 | 4.0 |  | 181 | 5.1 | 35 | 3.8 |  | 421 | 11.9 | 81 | 13.5 |
|  |  |  |  |  |  |  |  |  |  |  |  |  |  |  |  |  |  |  |  |
| Mode of contact | Telephone from patient, next of kin, caregiver (*n* = 19,895) | | | |  | Direct attendance  (*n* = 3197) | | | |  | Telephone from health professionals  (*n* = 12,480) | | | |  | EMCC or alarm (*n* = 2512) | | | |
| Men (*n* = 16,037) |  |  | 188 |  |  |  |  | 34 |  |  |  |  | 116 |  |  |  |  | 28 |  |
| 70-74 years | 2621 | (60.6) | 142 | Ref. |  | 617 | (14.3) | 34 | Ref. |  | 761 | (17.6) | 41 | Ref. |  | 329 | (7.6) | 18 | Ref. |
| 75-79 years | 2228 | (57.8) | 190 | 1.3 |  | 411 | (10.7) | 35 | 1.0 |  | 896 | (23.2) | 76 | .19 |  | 319 | (8.3) | 27 | 1.5 |
| 80-84 years | 1744 | (49.5) | 230 | 1.6 |  | 142 | (8.1) | 38 | 1.1 |  | 1186 | (33.7) | 157 | 3.8 |  | 308 | (8.7) | 41 | 2.3 |
| 85-89 years | 1099 | (41.1) | 262 | 1.8 |  | 52 | (5.3) | 34 | 1.0 |  | 1253 | (46.9) | 299 | 7.3 |  | 179 | (6.7) | 43 | 2.4 |
| ≥90 years | 548 | (33.1) | 285 | 2.0 |  |  | (3.1) | 27 | 0.8 |  | 966 | (58.3) | 502 | 12.0 |  | 92 | (5.5) | 48 | 2.7 |
|  |  |  |  |  |  |  |  |  |  |  |  |  |  |  |  |  |  |  |  |
| Women (*n* = 22,047) |  |  | 211 |  |  |  |  | 31 |  |  |  |  | 134 |  |  |  |  | 23 |  |
| 70-74 years | 3366 | (67.7) | 180 | Ref. |  | 596 | (12.0) | 32 | Ref. |  | 706 | (14.2) | 38 | Ref. |  | 301 | (6.1) | 16 | Ref. |
| 75-79 years | 2701 | (32.3) | 201 | 1.1 |  | 467 | (10.8) | 35 | 1.1 |  | 878 | (20.3) | 65 | 1.7 |  | 299 | (6.6) | 21 | 1.3 |
| 80-84 years | 2489 | (54.6) | 245 | 1.4 |  | 301 | (6.6) | 30 | 0.9 |  | 1493 | (32.8) | 147 | 3.9 |  | 273 | (6.0) | 27 | 1.7 |
| 85-89 years | 1843 | (42.0) | 238 | 1.3 |  | 220 | (5.0) | 28 | 0.8 |  | 2053 | (46.8) | 265 | 7.0 |  | 268 | (6.1) | 35 | 2.2 |
| ≥90 years | 1256 | (33.3) | 241 | 1.3 |  | 105 | (2.8) | 20 | 0.6 |  | 2288 | (60.1) | 439 | 12.0 |  | 155 | (4.1) | 30 | 1.9 |

**Additional file 2** Age and sex differences within mode of contact and first response initiated.

^1^Rate per 1000 inhabitants 70 years and older per year; ^2^Relative risk
